# Independent and Combined Effects of Resistance Training and Whey Protein on Skeletal Muscle Mass and Function in Individuals with MASLD Under Caloric Restriction

**DOI:** 10.3390/nu18010083

**Published:** 2025-12-26

**Authors:** Chae-Been Kim, Jinwoo Sung, Dohyun Ahn, Eun-Ah Jo, Kyung-Wan Baek, Hae-Ri Heo, Ju-Hwan Oh, Fengrui Zhang, Hyoung-Su Park, Hong-Soo Kim, Jung-Jun Park

**Affiliations:** 1 Department of Sport Science, Pusan National University, Busan 46241, Republic of Korea; chaebeen@pusan.ac.kr (C.-B.K.); sjw920412@pusan.ac.kr (J.S.); ahndo99@pusan.ac.kr (D.A.); jea0543@pusan.ac.kr (E.-A.J.); heohaeri@pusan.ac.kr (H.-R.H.); mtoto0417@pusan.ac.kr (J.-H.O.); zfree@pusan.ac.kr (F.Z.); 2 Research Institute of Human Ecology, Pusan National University, Busan 46241, Republic of Korea; 3 Department of Kinesiology and Sport Management, Texas Tech University, Lubbock, TX 79409, USA; 4 Research Institute of Pharmaceutical Sciences, Gyeongsang National University, Jinju 52828, Republic of Korea; baekgnu@gnu.ac.kr; 5 R&D Group, Maeil Health Nutrition Co., Ltd., Pyeongtaek 17714, Republic of Korea; parkhs@maeil.com; 6 Gastroenterology, Soon Chun Hyang University Cheonan Hospital, Cheonan 31151, Republic of Korea; khskhs@sch.ac.kr

**Keywords:** resistance training, whey protein supplementation, muscle mass, dietary control, MASLD

## Abstract

**Background/Objectives**: Metabolic dysfunction-associated steatotic liver disease (MASLD) often requires caloric restriction (CR) to reduce hepatic fat, but CR can compromise muscle mass and function. Resistance training and whey protein are anabolic stimuli, yet their independent and combined effects under substantial CR are unclear. **Methods**: In a 4-week randomized, double-blind trial, adults with MASLD (*n* = 45) under ~30% CR were allocated to four groups: resistance training with whey protein supplementation (E-PRO), resistance training with placebo (E-PLA), whey protein supplementation only (PRO), or placebo only (PLA). PRO groups consumed 1.5 g·kg^−1^·day^−1^ protein, while PLA groups consumed 0.8 g·kg^−1^·day^−1^. Exercise groups performed supervised exercise 5 days/week. Outcomes included body composition and isokinetic muscle function. **Results:** Across ~30% CR, all groups reduced body weight and fat mass without skeletal muscle mass loss; no between-group differences in body composition changes were observed. For peak torque, E-PRO improved across all regions except trunk, E-PLA improved except non-dominant shoulder flexors and trunk flexors, PRO improved non-dominant knee flexors and shoulder extensors, and PLA showed no change. For total work, E-PRO and E-PLA increased across all regions PRO improved dominant knee extensors/flexors and shoulder extensors; PLA increased only non-dominant knee extensors. E-PRO and E-PLA exceeded PLA for multiple knee/shoulder/trunk outcomes. **Conclusions**: In individuals with MASLD undergoing severe CR, ≥0.8 g·kg^−1^·day^−1^ of protein preserves skeletal muscle mass. However, the anabolic synergy of resistance training and protein for functional adaptation appears to be blunted by the substantial energy deficit.

## 1. Introduction

Metabolic dysfunction-associated steatotic liver disease (MASLD) is the most prevalent chronic liver condition worldwide and is associated with the risk of progression to hepatic inflammation, fibrosis, and eventually hepatocellular damage [1]. Therapeutic weight loss, particularly through significant caloric restriction (CR), remains the cornerstone strategy for reducing hepatic fat accumulation and improving liver-related outcomes [2].

However, while effective for hepatic improvement, CR produces a substantial energy deficit, thereby inducing a catabolic environment that threatens skeletal muscle homeostasis. Protein intake is a modifiable factor during weight loss, yet the degree to which commonly recommended intake levels are sufficient to support muscle mass and function under such metabolic stress remains unclear [3]. This question is particularly important in individuals with MASLD, who may experience anabolic resistance and muscle deterioration, potentially complicating adaptation to CR [4].

Recent evidence supports the clinical relevance of resistance training in MASLD. A systematic review of six randomized trials reported that resistance training significantly reduced hepatic fat and improved liver enzymes and insulin resistance [5]. However, these trials primarily focused on hepatic and metabolic outcomes, and muscle-related outcomes under a controlled CR remain less well investigated.

Resistance training and whey protein supplementation are potent anabolic strategies, as highlighted in a previous reviews [6]. Regular resistance exercise induces neuromuscular adaptations and promotes an anabolic environment that supports improvements in strength and muscle mass [7,8], while whey protein is a practical, leucine-rich option for supporting muscle protein synthesis [9]. Nonetheless, under conditions of substantial CR, reduced energy availability and altered substrate supply may constrain training adaptations and recovery following exercise [10].

While resistance training is clinically relevant for MASLD, muscle-related outcomes have been less consistently characterized than hepatic improvements [5]. Evidence from energy-deficit settings suggests that reduced energy availability during marked CR may limit lean mass accretion or shift adaptations toward maintenance rather than gain [11]. Interpretation of these muscle-related findings is further complicated by methodological limitations, including reliance on self-reported dietary intake, which lacks the precision to quantify intake in an energy-restricted state [12]. Building upon the methodological rigor of a prior tightly controlled feeding study [13], this study aimed to further clarify the independent and combined effects of whey protein supplementation and resistance training within the specific context of a significant CR.

Therefore, the primary objective of this study was to assess the independent and combined effects of whey protein supplementation and resistance training on skeletal muscle mass preservation and muscle function in individuals with MASLD undergoing a strictly controlled CR. We sought to clarify whether whey protein provides additional muscle-related benefits beyond resistance training alone under strict CR.

## 2. Materials and Methods

### 2.1. Participants

Forty-five adults (aged 20–69 years) residing in the Republic of Korea were enrolled. All participants received a full explanation of study procedures and provided written informed consent. Inclusion criteria were as follows: (i) men or women aged 20–69 years; (ii) a confirmed diagnosis of MASLD according to established clinical guidelines [14]; (iii) stable body weight (±2 kg) for ≥3 months prior to enrollment; and (iv) no regular exercise > 30 min/session on >2 days/week during the preceding 3 months. All participants were cleared for exercise using the PAR-Q+ and a physician’s clinical assessment of exercise readiness. Exclusions included known cardiovascular or renal disease, current use of medications likely to influence study outcomes, current smoking, milk allergy or lactose intolerance, and pregnancy or lactation.

Participants were recruited between March and August 2024. Recruitment was closed due to operational constraints within the study timeline. The a priori sample size (N = 80; 20 per group) was determined for the trial’s primary hepatic endpoint, which will be reported in a separate manuscript, assuming α = 0.05, and power = 0.85, and 30% attrition. No separate a priori power calculation was performed specifically for the muscle-related outcomes examined in this study.

### 2.2. Study Design

A computer-generated simple randomization list assuming equal allocation to the four groups was created by a researcher independent of outcome assessment and intervention delivery. Sequential allocations were applied as participants enrolled, and recruitment was closed before the full randomization list had been exhausted, resulting in unequal realized group sizes: resistance exercise with whey protein supplementation (E-PRO, *n* = 14), resistance exercise with placebo (E-PLA, *n* = 12), whey protein supplementation only (PRO, *n* = 11), or placebo only (PLA, *n* = 8).

The trial was double-blind for supplementation; participants, investigators, and outcome assessors were blinded to whey versus placebo assignment. The whey and placebo products were organoleptically indistinguishable (identical appearance, taste, and packaging). Exercise allocation could not be masked but was implemented according to the randomized assignment. Outcomes were assessed over the 4-week intervention period. All participants were instructed to avoid additional structured exercise and to consume only the provided meals for the duration of the study. The intervention duration was set to 4 weeks to align with the practical constraints of providing strictly controlled, fully provided meals, and to focus specifically on early muscle function and mass responses during the initial phase of CR [15].

The protocol was approved by the Institutional Review Board of Pusan National University (IRB No. 2023_229_HR; approval date: 26 December 2023) and preregistered with the Clinical Research Information Service (KCT0009143; registration date: 30 January 2024) prior to first participant enrollment. This study was conducted in accordance with the Declaration of Helsinki of 1975, revised in 2013.

### 2.3. Intervention

#### 2.3.1. Whey Protein Supplement and Placebo

Whey protein supplement and placebo were provided in powdered form (Maeil Health Nutrition, Pyeongtaek, Republic of Korea) in indistinguishable packaging to maintain double-blinding. Participants assigned to protein supplementation (E-PRO, PRO) targeted a total daily protein intake of 1.5 g·kg^−1^, comprising 0.8 g·kg^−1^ from standardized meals and 0.7 g·kg^−1^ from the whey protein supplement. The total protein amount for the whey protein supplementation groups was selected to represent a practical, evidence-informed high-protein condition during CR, providing a clear contrast with an RDA level intake [16,17].

Participants in the placebo groups (E-PLA, PLA) consumed an amount of placebo powder isovolumetric and schedule-matched to the whey supplement and obtained 0.8 g·kg^−1^·day^−1^ protein from standardized meals, consistent with general dietary recommendations. To align with per-meal muscle protein synthesis threshold and the diminishing returns observed with large single boluses of protein, the daily supplement dose (0.7 g·kg^−1^·day^−1^) was divided into five servings and consumed at evenly spaced intervals across the day [18].

#### 2.3.2. Dietary Control

Each participant’s daily energy intake target was set at 70% of total daily energy expenditure (TDEE), calculated as resting metabolic rate (RMR) × physical activity level (PAL) [19]. This level was selected to induce a clinically meaningful early weight-loss target relevant to improving hepatic steatosis and is consistent with prior NAFLD/MASLD dietary intervention applying ~25–30% energy restriction or comparable daily deficits [20,21]. A relative restriction based on TDEE was used to standardize the energy deficit across individuals rather than using a fixed absolute kcal reduction. For the exercise groups (E-PRO and E-PLA), PAL was adjusted to account for the additional energy expended during training sessions.

Total calories were allocated as follows: 25% from fat; protein (or matched placebo volume) was then accounted for; the remainder was provided as carbohydrate. Customized meals were delivered three times per day—breakfast by Togosalad (Brosgo Inc., Seoul, Republic of Korea) and lunch/dinner by Hansot Inc. (Seoul, Republic of Korea)—and were formulated to meet individual macronutrient targets. Daily verification of energy and macronutrient content was performed using the providers’ nutrition reports.

Dietary adherence was monitored through participant-submitted meal videos and real-time food-intake diaries. Participants were instructed to consume only the provided meals; any deviations from the prescribed diet were recorded immediately in the diary and reported to investigators. For each participant, average daily energy intake over the 4-week intervention was divided by the prescribed caloric target to derive an adherence ratio.

#### 2.3.3. Resistance Training Protocol

Participants in the exercise groups (E-PRO and E-PLA) completed a supervised resistance-training program 5 days/week for 4 weeks, with each session lasting 60 min (10 min warm-up, 40 min resistance training, 10 min cool-down). Training intensity progressed weekly from 50% of one-repetition maximum (1-RM) in week 1 to 60%, 70%, and 75% 1-RM in weeks 2–4, respectively. Exercises targeted three major muscle regions—chest, back, and lower extremities—each trained twice per week [8]. The exercise selection included standard machine and free-weight movements such as chest press/machine fly, latissimus dorsi pulldown/seated row, shoulder press/dumbbell lateral raises, and lower-body exercises including leg press, leg extension, and leg curl. For each exercise, participants performed 4 sets (including one warm-up set) of 8–12 repetitions with 60–120 s of inter-set rest [22]. Core/abdominal exercises, including planks and crunch variations, were performed daily. All sessions were conducted under the supervision of qualified exercise professionals, and participants were instructed to abstain from additional structured physical activity beyond the prescribed program for the duration of the intervention. Exercise adherence was monitored using attendance records and quantified as the percentage of completed supervised sessions out of the 20 scheduled sessions.

### 2.4. Measurements

#### 2.4.1. Body Composition

Participants arrived in the morning after fasting for ≥8 h, abstaining from caffeine for ≥12 h, and abstaining from alcohol and vigorous exercise for ≥24 h; they voided prior to testing. Body composition was assessed using a multi-frequency bioelectrical impedance analyzer (InBody BWA 2.0; InBody Corp., Seoul, Republic of Korea) with standard 8-point electrode placement. Two consecutive measurements were obtained by the same trained assessor, and the mean value was used for analysis.

#### 2.4.2. Isokinetic Muscle Function Assessment

Muscle function was evaluated using an isokinetic dynamometer (Cybex770; CSMi, Stoughton, MA, USA). Peak torque and total work of flexors and extensors were assessed for the knee, shoulder, and trunk. Ranges of motion were set to 0–90° (knee), 0–135° (shoulder), and −15–95° (trunk). Dominant and non-dominant limbs were both tested for the knee and shoulder; the order of side testing was randomized. Participants were stabilized with straps according to manufacturer guidelines.

Maximal strength was tested at 60°·s^−1^. After three familiarization trials, participants performed five maximal repetitions; peak torque was defined as the highest value across repetitions and normalized to body mass (Nm·kg^−1^ × 100). Endurance was tested at 180°·s^−1^. Following three warm-up trials, participants completed 15 repetitions; total work was calculated as the sum across repetitions and expressed relative to body mass (J·kg^−1^ × 100). Rest between sets was standardized at 60–120 s.

#### 2.4.3. Resting Metabolic Rate

RMR was measured by indirect calorimetry (Quark b^2^, COSMED, Albano Laziale (RM), Italy) using a ventilated-canopy system. After an ≥8 h fast, participants rested supine for 20 min in a quiet, thermoneutral room before measurement. Gas exchange was recorded for 15 min; the first 5 min were discarded for stabilization, and the subsequent 10 min were averaged to compute RMR using the Weir equation. The system was calibrated according to the manufacturer’s instructions before each testing session.

#### 2.4.4. Physical Activity Level

Baseline PAL was estimated using the Korean physical activity classification table [19]. Time (min·day^−1^) spent in each activity was multiplied by the corresponding MET value to estimate activity energy expenditure; PAL was then derived and used with measured RMR to calculate TDEE. For exercise groups, PAL was adjusted to incorporate the supervised training energy.

#### 2.4.5. 1-RM Assessment

1-RM testing was conducted using the same movements as in the training program. After a standardized warm-up, loads were selected to induce failure within ≤10 repetitions. Predicted 1-RM was calculated using the Brzycki Equation (1) [23]:

(1) $$Predicted\;1\text{-}RM=\frac{Weight\;lifted}{1.0278-(0.0278\times repetitions)}$$

### 2.5. Data Analysis

Analyses used a complete-case approach, including all randomized participants with available pre- and post-intervention data for each outcome. Missing values were not imputed; analyses were performed on complete cases. Analyses were performed in SPSS v29 (IBM, Armonk, NY, USA). Within-group normality was assessed using the Shapiro–Wilk test; variables violating normality assumptions were analyzed using nonparametric procedures as pre-specified. Homogeneity of variances was evaluated with Levene’s test.

Within-group pre–post changes were assessed using the Wilcoxon signed-rank test. Between-group differences in change scores were tested with the Kruskal–Wallis test, followed, if significant, by Bonferroni-adjusted pairwise comparisons (adjusted α = 0.0083, based on six pairwise tests). To aid interpretation, effect sizes were reported for all primary analyses and are reported in the relevant tables. Statistical significance was set at *p* < 0.05.

## 3. Results

### 3.1. Participant Characteristics

The baseline characteristics of the participants are shown in Table 1. Three participants discontinued the intervention. Although realized group sizes were unequal, baseline characteristics were comparable across groups. Baseline hepatic steatosis, assessed by the controlled attenuation parameter (CAP), also did not differ significantly between groups, and the CAP-based steatosis grade distribution indicated that most participants were classified as S3, with smaller numbers in S1 and S2. Across all participants, mean energy intake was 100.3 ± 2.0% of the prescribed caloric target (70% of TDEE), indicating good adherence to the intended energy restriction. Among completers, attendance for supervised resistance-training sessions was 100% (20/20 sessions).

### 3.2. Body Weight and Body Composition

The changes in body weight and bioelectrical impedance analysis (BIA)-estimated body composition before and after the intervention are shown in Table 2. All four groups showed significant reductions in body weight and body fat mass, whereas skeletal muscle mass did not change significantly. No between-group differences were found for changes in any body composition variable, which was reflected by negligible-to-small effect sizes across all comparisons (0.000–0.052).

### 3.3. Isokinetic Muscle Function

#### 3.3.1. Normalized Peak Torque

**Figure 1:**
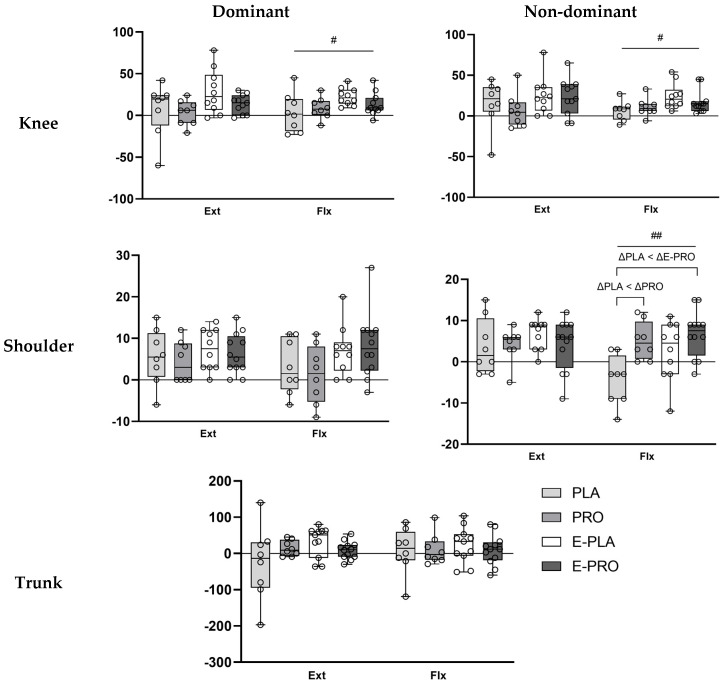
Changes in normalized peak torque (Nm·kg^−1^ × 100) of dominant and non-dominant knee and shoulder, and trunk extensors and flexors. Data represent the changes in peak torque normalized to body weight (BW) and expressed as Nm·kg^−1^ × 100. # *p* < 0.05, ## *p* < 0.01 indicate statistically significant group differences (Kruskal–Wallis test).

Figure 1 displays changes in peak torque normalized to body weight (Nm·kg^−1^ × 100) for the dominant and non-dominant knee and shoulder joints, as well as the trunk. In the non-dominant shoulder flexors, the E-PRO (*p* < 0.001) and PRO (*p* = 0.005) groups showed significantly greater increases than the PLA group. A significant group effect was found for changes in normalized peak torque of the dominant (*p* = 0.047) and non-dominant (*p* = 0.030) knee flexors. However, post hoc pairwise comparisons did not detect any statistically significant differences between groups.

The E-PRO group demonstrated significant increases in Nm·kg^−1^ × 100 across all measured muscle groups except the trunk, supported by substantial effect sizes (Table 3). In the E-PLA group, improvements were observed in all regions except the non-dominant shoulder flexors and the trunk flexors. The PRO group showed significant increases in the non-dominant knee flexors and non-dominant shoulder extensors. No significant changes were observed in any measured muscle groups in the PLA group.

#### 3.3.2. Normalized Total Work

**Figure 2:**
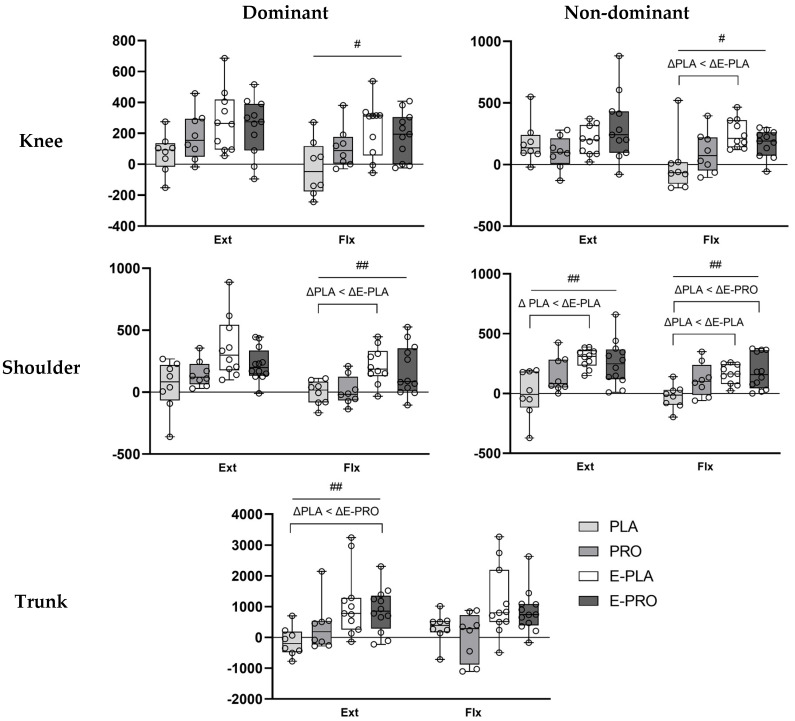
Changes in normalized total work (J·kg^−1^ × 100) of dominant and non-dominant knee and shoulder, and trunk extensors and flexors. Data represent the changes in total work values normalized to body weight (BW) and expressed as J·kg^−1^ × 100. # *p* < 0.05, ## *p* < 0.01 indicate statistically significant group differences (Kruskal–Wallis test).

Changes in total work normalized to body weight (J·kg^−1^ × 100) for the knee and shoulder joints and the trunk are shown in Figure 2. In the E-PRO group, the increase in normalized total work was significantly greater than in the PLA group for the non-dominant shoulder flexors (*p* = 0.003) and trunk extensors (*p* = 0.003). In the E-PLA group, significantly greater increases than the PLA group were observed in the non-dominant knee flexors (*p* = 0.006), dominant shoulder flexors (*p* = 0.002), non-dominant shoulder extensors and flexors (both *p* < 0.001), and trunk extensors (*p* = 0.002). A significant group effect was found for changes in normalized total work of the dominant knee extensors (*p* = 0.037) and dominant shoulder extensors (*p* = 0.031), but post hoc pairwise comparisons did not reveal any statistically significant differences between groups.

The E-PRO and E-PLA groups showed significant increases in J·kg^−1^ × 100 across all measured muscle groups (Table 4). Notably, these functional improvements were supported by predominantly large effect sizes across most muscle groups. The PRO group exhibited significant improvements in the dominant knee extensors and flexors, as well as in the dominant and non-dominant shoulder extensors. In contrast, the PLA group showed a significant increase only in the non-dominant knee extensors.

## 4. Discussion

A key observation of this study is that skeletal muscle mass using BIA was maintained across all intervention groups despite a 30% CR. Although loss of skeletal muscle mass is commonly reported during weight-loss interventions, particularly over longer durations [25,26], such reductions were not detected in this 4-week trial, which may reflect the relatively short period and the tightly controlled dietary provision. Because all groups consumed at least ≥0.8 g·kg^−1^·day^−1^, adequate protein intake may have contributed to limiting short-term lean-mass loss under energy restriction. Importantly, BIA-derived estimates can be influenced by hydration status and related changes during CR, which may limit sensitivity for detecting small changes in skeletal muscle mass. Therefore, these findings should be interpreted cautiously. Moreover, baseline adiposity may have influenced the susceptibility to muscle catabolism during CR. Because our cohort largely represented overweight to mild obesity, these findings may not directly generalize to individuals with higher or severe obesity, where pronounced anabolic resistance could exacerbate the risk of lean mass loss. Numerous studies indicate that intakes above the current RDA more effectively preserve muscle during weight loss [26,27,28], but in this context, the absence of measurable loss should not be interpreted as evidence that 0.8 g·kg^−1^·day^−1^ is optimal. Accordingly, although preservation observed in this study is encouraging, it would be premature to generalize 0.8 g·kg^−1^·day^−1^ as optimal across weight-loss contexts.

Participants assigned to resistance training exhibited significant improvements in both muscle strength and muscular endurance. Given the 4-week intervention, these functional gains are most consistent with early neural adaptations rather than hypertrophic adaptations [7,29]. Prior work indicates that early strength gains can occur without structural changes via improved neural control [7,8], reflected in enhanced motor-unit recruitment and synchronization [4,29]. The observed gains in muscular endurance are likewise consistent with adequate mechanical loading from a program of five sessions per week of 8–12 repetitions across four sets, potentially facilitated by neural mechanisms [7,22]. Although direct indices of neural activation were not collected, the short intervention period supports the interpretation that early functional improvements primarily reflect enhanced neuromuscular efficiency rather than muscle hypertrophy [7,8].

Strength gains were particularly pronounced at the shoulder joint. Such region-specific responses are frequently observed in the early phase of resistance training novices, and may reflect greater upper-body responsiveness, potentially due to lower baseline strength and/or lower habitual use relative to the lower body, together with early neural adaptation [30]. Nevertheless, differences in responsiveness between upper- and lower-body muscle groups may also involve intrinsic physiological factors (e.g., androgen receptor density) [29]. The present data do not allow us to determine whether the observed response is driven by baseline performance differences or by such intrinsic properties.

Related to these upper-body findings, the protein-only group showed significantly greater improvements in non-dominant shoulder flexor function compared with the placebo group. However, this pattern was not observed in the corresponding extensor muscles or on the dominant side, suggesting that the response was localized and not consistent across related outcomes. Therefore, this shoulder-specific response should be interpreted with caution and may, at least in part, reflect variability rather than a consistent physiological effect.

Notably, the combined intervention (resistance training plus protein) did not confer consistent additional benefits over single-modality interventions for strength or muscular endurance. This contrasts with some prior findings of positive interaction effects between resistance training and protein supplementation [6,13,31]. Under marked energy restriction, it has been suggested that altered substrate availability and activation of energy-sensing pathways may attenuate anabolic adaptations to resistance training [32]. However, we did not measure muscle glycogen or molecular markers of anabolic signaling in this trial, so these potential mechanisms remain speculative and cannot be confirmed by our data.

## 5. Limitations

This study has several limitations. Firstly, the 4-week intervention period was appropriate for assessing early changes and short-term muscle preservation during CR, but it may be insufficient to detect hypertrophy or longer-term functional changes between interventions. Secondly, the sample size was determined for the primary hepatic endpoint rather than the muscle outcomes analyzed here. Therefore, the present study may have been underpowered to detect small-to-moderate between-group differences in skeletal muscle mass and function, increasing the risk of type II error. We also acknowledge that the modest and unequal group sizes, which were not optimized for muscle-related endpoints, are not sufficient to support adequately powered subgroup analyses, and such analyses will need to be addressed in larger future trials. In addition, recruitment did not reach the planned target, likely due to stringent eligibility criteria and the intensive intervention requirements. Consequently, participants may represent a more adherent and health-motivated subset of the target population, which may limit the generalizability of these findings. Third, we did not assess mechanistic or mediating markers, limiting our ability to explain the observed responses. Fourthly, sex-stratified analyses were not performed due to the limited sample size. Therefore, potential sex-specific responses to CR and resistance training warrant investigation in larger trials. Finally, because participants were overweight to mildly obese, our findings may not generalize to individuals with more severe obesity or to weight-loss contexts with different degrees of energy restriction or protein prescriptions. Despite these limitations, the randomized controlled design, double-blind supplementation, clinically relevant MASLD population, and tightly controlled dietary provision strengthen internal validity and practical relevance.

## 6. Conclusions

In this 4-week RCT in individuals with MASLD undergoing intentional weight loss under marked energy restriction, skeletal muscle mass was maintained across all groups, while resistance training was associated with broader improvements in isokinetic muscle function compared with non-exercise conditions. Compared with placebo (dietary protein: 0.8 g·kg^−1^·day^−1^), whey supplementation increased total protein intake to 1.5 g·kg^−1^·day^−1^, but did not consistently provide additional improvements in isokinetic muscle function beyond the effects of resistance training. These findings should be interpreted within the specific context of MASLD, a substantial calorie deficit, and a short intervention duration. Clinically, prioritizing structured resistance training while ensuring adequate daily protein intake may be a pragmatic strategy to support the maintenance of lean mass and muscle function during rapid weight loss. Further studies are needed to determine whether additive benefits emerge under less severe energy restrictions, different protein prescriptions, or longer intervention periods.

## Figures and Tables

**Table 1:** Participants’ characteristics at the baseline.

Factor	PLA (*n* = 8)	PRO (*n* = 8)	E-PLA (*n* = 11)	E-PRO (*n* = 12)
Age (years)	30 ± 7	40 ± 15	33 ± 14	34 ± 10
Male/Female (*n*)	4/4	4/4	5/6	6/6
Height (cm)	168 ± 9	170 ± 10	172 ± 8	166 ± 8
Body weight (kg)	74.9 ± 13.1	83.0 ± 15.8	81.7 ± 8.8	80.6 ± 15.6
BMI (kg/m^2^)	26.2 ± 2.5	28.6 ± 3.4	27.5 ± 2.0	29.1 ± 4.0
CAP (dB/m)	281.3 ± 20.1	300.4 ± 29.7	302.0 ± 41.2	306.3 ± 37.7
Steatosis grade S1/S2/S3 (*n*)	2/2/4	1/1/6	3/1/6	1/3/8
RMR (kcal/day)	2004.5 ± 352.3	1705.8 ± 344.4	1667.1 ± 383.4	1707.5 ± 371.8
TDEE (kcal/day)	3032.0 ± 465.6	2561.5 ± 573.4	2318.8 ± 389.8	2390.4 ± 585.2

Values are presented as mean ± standard deviation or *n*. Steatosis grade was defined using CAP cutoffs (S2: 248–267, S2: 268–279, S3: ≥280 dB/m) as previously reported [24]. CAP was missing for one participant in the E-PLA group (*n* = 10); therefore, steatosis grade could not be assigned. PLA, placebo; PRO, protein supplementation; E-PLA, exercise with placebo; E-PRO, exercise combined with protein supplementation; BMI, body mass index; CAP, controlled attenuation parameter; RMR, resting metabolic rate; TDEE, total daily energy expenditure.

**Table 2:** Changes in body weight and body composition.

Variable	Group	Baseline	Week 4	Changes	Kruskal–Wallis H Test		
					χ^2^	*p*	ε^2^
Body weight (kg)	PLA	74.9 ± 13.1	72.5 ± 12.9 *	−2.3 ± 1.6	0.693	0.875	0.000
	PRO	83.0 ± 15.8	80.3 ± 14.3 *	−2.7 ± 2.5			
	E-PLA	81.7 ± 8.8	79.0 ± 8.4 **	−2.7 ± 1.6			
	E-PRO	80.6 ± 15.6	78.1 ± 14.8 **	−2.4 ± 1.6			
Body fat mass (kg)	PLA	24.8 ± 4.4	22.9 ± 4.0 *	−1.9 ± 1.4	4.836	0.184	0.052
	PRO	28.9 ± 8.0	26.8 ± 8.9 *	−2.1 ± 2.2			
	E-PLA	27.3 ± 3.8	24.4 ± 3.5 **	−3.0 ± 1.2			
	E-PRO	27.3 ± 6.6	24.6 ± 5.9 **	−2.7 ± 1.4			
Skeletal muscle mass (kg)	PLA	27.6 ± 6.3	27.4 ± 6.2	−0.2 ± 0.4	4.499	0.212	0.043
	PRO	30.1 ± 8.7	29.8 ± 8.9	−0.4 ± 0.8			
	E-PLA	30.4 ± 5.5	30.6 ± 5.8	0.2 ± 0.8			
	E-PRO	29.7 ± 6.7	30.0 ± 6.8	0.3 ± 0.4			

Values are presented as mean ± standard deviation. PLA—placebo; PRO—protein supplementation; E-PLA—exercise with placebo; E-PRO—exercise combined with protein supplementation. * *p* < 0.05, ** *p* < 0.01 indicate statistically significant differences based on the Wilcoxon signed rank test.

**Table 3:** Changes in peak torque normalized to body weight (Nm·kg^−1^ × 100).

			Group	Pre	Post	Wilcoxon		
						Z	*p*	Effect Size (*r*)
Knee	Do	Ext	PLA	156.88 ± 22.62	164.00 ± 36.57	−1.053	0.292	0.372
			PRO	149.25 ± 57.44	152.50 ± 58.78	−0.561	0.574	0.198
			E-PLA	143.30 ± 28.05	171.70 ± 40.37	−2.547	0.001	0.805
			E-PRO	141.80 ± 34.50	156.80 ± 35.62	−2.549	0.011	0.806
		Flx	PLA	104.38 ± 21.12	107.88 ± 26.07	−0.254	0.799	0.090
			PRO	89.12 ± 37.62	97.75 ± 44.34	−1.690	0.091	0.598
			E-PLA	91.70 ± 20.92	114.20 ± 20.60	−2.805	0.005	0.887
			E-PRO	85.60 ± 19.05	100.50 ± 17.58	−2.807	0.005	0.888
	Non-do	Ext	PLA	150.13 ± 25.75	165.50 ± 36.21	−1.400	0.161	0.495
			PRO	153.38 ± 59.71	160.38 ± 62.69	−0.702	0.483	0.248
			E-PLA	143.00 ± 39.98	167.80 ± 48.54	−2.521	0.012	0.797
			E-PRO	141.80 ± 34.47	168.10 ± 36.36	−2.604	0.009	0.823
		Flx	PLA	99.63 ± 19.37	105.63 ± 19.85	−1.355	0.176	0.479
			PRO	90.62 ± 34.65	101.38 ± 39.40	−2.198	0.028	0.777
			E-PLA	87.50 ± 22.12	111.60 ± 24.74	−2.805	0.005	0.887
			E-PRO	89.50 ± 17.62	108.10 ± 22.55	−2.814	0.005	0.890
Shoulder	Do	Ext	PLA	71.25 ± 11.90	76.75 ± 13.69	−1.778	0.075	0.629
			PRO	72.38 ± 14.08	76.75 ± 17.19	−1.826	0.068	0.646
			E-PLA	68.50 ± 12.34	75.80 ± 15.44	−2.677	0.007	0.847
			E-PRO	65.50 ± 10.61	71.83 ± 12.32	−2.812	0.005	0.812
		Flx	PLA	64.38 ± 8.26	67.50 ± 11.03	−1.265	0.206	0.447
			PRO	66.25 ± 9.42	67.50 ± 12.93	−0.509	0.611	0.180
			E-PLA	63.50 ± 9.79	70.60 ± 9.90	−2.536	0.011	0.802
			E-PRO	58.50 ± 12.76	66.58 ± 9.38	−2.720	0.007	0.785
	Non-do	Ext	PLA	70.75 ± 10.90	74.50 ± 13.77	−1.378	0.168	0.487
			PRO	70.13 ± 12.72	74.25 ± 13.58	−2.046	0.041	0.723
			E-PLA	67.60 ± 15.10	74.40 ± 15.20	−2.692	0.007	0.851
			E-PRO	68.33 ± 11.26	72.50 ± 12.28	−2.014	0.044	0.581
		Flx	PLA	64.75 ± 11.51	60.38 ± 11.28	−1.725	0.084	0.610
			PRO	55.88 ± 9.75	61.00 ± 12.74	−2.214	0.027	0.783
			E-PLA	59.70 ± 12.28	62.30 ± 8.96	−1.131	0.258	0.358
			E-PRO	55.25 ± 9.78	62.00 ± 7.60	−2.728	0.006	0.788
Trunk	Ext		PLA	244.25 ± 30.88	252.00 ± 50.44	−0.676	0.499	0.239
			PRO	201.88 ± 68.92	216.00 ± 64.21	−1.524	0.128	0.539
			E-PLA	216.45 ± 52.44	248.91 ± 53.92	−2.047	0.041	0.617
			E-PRO	204.42 ± 49.06	213.33 ± 39.96	−1.179	0.239	0.340
	Flx		PLA	266.63 ± 39.40	290.50 ± 54.10	−1.540	0.123	0.544
			PRO	225.38 ± 42.23	266.25 ± 70.59	−0.350	0.726	0.124
			E-PLA	257.00 ± 48.67	280.55 ± 24.25	−1.327	0.185	0.400
			E-PRO	261.58 ± 62.55	271.58 ± 42.50	−0.864	0.387	0.249

Values are presented as mean ± standard deviation. Peak torque values were normalized to body weight and expressed as Nm·kg^−1^ × 100, calculated as (peak torque ÷ body weight) × 100. Within-group pre-post comparisons were performed using the Wilcoxon signed-rank test, and Z statistics and *p* values are reported. Effect size r was calculated as $\left|Z\right|/\sqrt{N}$. Sample sizes varied by joint as follows: for the knee, PLA (*n* = 8), PRO (*n* = 8), E-PLA (*n* = 10), E-PRO (*n* = 10); for the shoulder, PLA (*n* = 8), PRO (*n* = 8), E-PLA (*n* = 10), E-PRO (*n* = 12); and for the trunk, PLA (*n* = 8), PRO (*n* = 8), E-PLA (*n* = 11), E-PRO (*n* = 12). PLA—placebo; PRO—protein supplementation; E-PLA—exercise with placebo; E-PRO—exercise combined with protein supplementation.

**Table 4:** Changes in total work normalized to body weight (J·kg^−1^ × 100).

			Group	Pre	Post	Wilcoxon		
						Z	*p*	Effect Size (*r*)
Knee	Do	Ext	PLA	1809.88 ± 296.17	1880.00 ± 324.43	−1.400	0.161	0.495
			PRO	1573.75 ± 614.41	1756.13 ± 642.41	−2.380	0.017	0.841
			E-PLA	1564.70 ± 457.91	1846.00 ± 439.57	−2.803	0.005	0.886
			E-PRO	1566.10 ± 407.94	1839.20 ± 458.43	−2.701	0.007	0.854
		Flx	PLA	1420.88 ± 295.44	1394.88 ± 367.40	−0.350	0.726	0.124
			PRO	1210.38 ± 446.94	1319.00 ± 500.54	−2.028	0.043	0.717
			E-PLA	1232.30 ± 326.91	1464.20 ± 328.99	−2.497	0.013	0.790
			E-PRO	1131.50 ± 328.87	1325.10 ± 369.77	−2.310	0.021	0.730
	Non-do	Ext	PLA	1651.00 ± 339.87	1829.38 ± 303.51	−2.380	0.017	0.841
			PRO	1624.25 ± 596.59	1721.50 ± 554.31	−1.680	0.093	0.594
			E-PLA	1576.50 ± 476.17	1770.90 ± 488.76	−2.803	0.005	0.886
			E-PRO	1524.10 ± 436.79	1866.30 ± 419.03	−2.803	0.005	0.886
		Flx	PLA	1386.63 ± 277.86	1383.13 ± 250.42	−0.980	0.327	0.346
			PRO	1126.88 ± 388.60	1227.25 ± 443.20	−1.521	0.128	0.538
			E-PLA	1200.30 ± 302.20	1451.10 ± 338.51	−2.803	0.005	0.886
			E-PRO	1184.90 ± 315.44	1356.10 ± 246.88	−2.701	0.007	0.854
Shoulder	Do	Ext	PLA	1411.25 ± 323.81	1461.88 ± 424.65	−0.980	0.327	0.346
			PRO	1387.38 ± 456.08	1536.00 ± 448.92	−2.521	0.012	0.891
			E-PLA	1211.00 ± 525.66	1572.40 ± 446.89	−2.803	0.005	0.886
			E-PRO	1335.50 ± 332.01	1558.33 ± 373.48	−2.981	0.003	0.861
		Flx	PLA	839.75 ± 282.49	834.25 ± 258.81	0.000	0.999	0.000
			PRO	979.38 ± 431.65	991.00 ± 427.36	−0.070	0.944	0.025
			E-PLA	791.20 ± 338.28	1005.50 ± 335.21	−2.701	0.007	0.854
			E-PRO	963.17 ± 290.59	1136.75 ± 324.56	−2.312	0.021	0.667
	Non-do	Ext	PLA	1326.88 ± 321.44	1324.75 ± 472.97	−0.140	0.889	0.049
			PRO	1283.38 ± 512.88	1440.13 ± 419.61	−2.366	0.018	0.837
			E-PLA	1225.00 ± 415.91	1519.70 ± 443.43	−2.803	0.005	0.886
			E-PRO	1277.00 ± 386.48	1532.67 ± 318.19	−3.059	0.002	0.883
		Flx	PLA	738.88 ± 279.08	713.13 ± 303.25	−0.631	0.528	0.223
			PRO	744.25 ± 378.40	859.50 ± 363.52	−1.960	0.050	0.693
			E-PLA	723.60 ± 270.42	883.60 ± 277.34	−2.803	0.005	0.886
			E-PRO	792.33 ± 246.88	973.83 ± 261.52	−2.936	0.003	0.848
Trunk	Ext		PLA	2658.88 ± 1374.10	2521.13 ± 1500.95	−0.840	0.401	0.297
			PRO	1791.75 ± 1373.18	2153.88 ± 1945.41	−1.120	0.263	0.396
			E-PLA	1721.91 ± 1140.03	2816.27 ± 1322.86	−2.756	0.006	0.831
			E-PRO	1592.92 ± 864.60	2472.25 ± 930.43	−2.746	0.006	0.793
	Flx		PLA	2484.13 ± 967.24	2798.62 ± 1046.40	−1.542	0.123	0.545
			PRO	2825.50 ± 1525.78	2839.38 ± 1776.86	−0.140	0.889	0.049
			E-PLA	2430.55 ± 1050.80	3560.27 ± 1239.55	−2.756	0.006	0.831
			E-PRO	2356.92 ± 824.01	3204.17 ± 932.88	−2.981	0.003	0.861

Values are presented as mean ± standard deviation. Total work values were normalized to body weight and expressed as J·kg^−1^ × 100, calculated as (total work ÷ body weight) × 100. Within-group pre-post comparisons were performed using the Wilcoxon signed-rank test, and Z statistics and *p* values are reported. Effect size r was calculated as $\left|Z\right|/\sqrt{N}$. Sample sizes varied by joint as follows: for the knee, PLA (*n* = 8), PRO (*n* = 8), E-PLA (*n* = 10), E-PRO (*n* = 10); for the shoulder, PLA (*n* = 8), PRO (*n* = 8), E-PLA (*n* = 10), E-PRO (*n* = 12); and for the trunk, PLA (*n* = 8), PRO (*n* = 8), E-PLA (*n* = 11), E-PRO (*n* = 12). PLA—placebo; PRO—protein supplementation; E-PLA—exercise with placebo; E-PRO—exercise combined with protein supplementation.

## Data Availability

The data are not publicly available due to privacy. The data that support the findings of this study are available from the corresponding author (J.-J.P.), upon reasonable request.

## Abbreviations

The following abbreviations are used in this manuscript:

BMI	Body mass index
CR	Caloric restriction
MASLD	Metabolic dysfunction-associated steatotic liver disease
PAL	Physical activity level
RDA	Recommended dietary allowance
RMR	Resting metabolic rate
TDEE	Total daily energy expenditure
1-RM	One-repetition maximum
